# Central Retinal Artery Occlusion Associated with Sildenafil Overdose

**DOI:** 10.1155/2021/2006271

**Published:** 2021-09-04

**Authors:** Mojtaba Abrishami, Seyedeh Maryam Hosseini, Hamid Mohseni, Majid Razavi, Amir Ghaffarian Mashhadi Nejad, Mohammad Baghi Yazdi, Ghodsieh Zamani

**Affiliations:** ^1^Eye Research Center, Mashhad University of Medical Sciences, Mashhad, Iran; ^2^Department of Anesthesiology, School of Medicine, Mashhad University of Medical Sciences, Mashhad, Iran

## Abstract

**Background:**

To report a patient with central retinal artery occlusion (CRAO) associated with sildenafil overdose. *Case Presentation*. A forty-two-year-old male presented three hours after sudden painless visual loss in the right eye. BCVA was counting finger in two meters, and relative afferent pupillary defect was positive. Fundus examination revealed retinal whiteness except in a limited area of papillomacular bundle and cherry red spot. He consumed two 100 mg film-coated sildenafil tablet (Vizarsin, Krka, d.d., Novo mesto, Slovenia) twelve hours apart, and the last one was six hours before visual loss. He was diagnosed with CRAO with cilioretinal artery sparing. Although we did not find any emboli, anterior chamber paracentesis was done. Four weeks later, BCVA improved to 20/80, with resolving of retinal edema. Cardiovascular, carotid arteries, and neurologic evaluations were negative for any predisposing factor.

**Conclusion:**

CRAO is a vision threatening condition that might be associated with the overdose of sildenafil.

## 1. Background

Sildenafil is a selective phosphodiesterase (PDE) type 5 inhibitor. It usually acts via regulation of vascular tone and relaxing vascular musculature [1]. By increasing the nitric oxide/cyclic guanosine monophosphate pathway, it is helpful in treating erectile dysfunction and pulmonary arterial hypertension [2]. The most common adverse events reported by patients using sildenafil have been mild to moderate transient headache, facial flushing, rhinitis, dyspepsia, and abnormal vision, usually bluish discoloration and impaired blue/green discrimination [3].

Central retinal artery occlusion (CRAO), as an ophthalmic emergency, is an ocular analogue of cerebral stroke. CRAO is considered as an end-organ ischemia and often associated with underlying atherosclerotic diseases [4]. Here, we present an otherwise healthy young man presented CRAO with cilioretinal artery sparing, who overdosed sildenafil with total 200 mg consumption in twelve hours.

## 2. Case Presentation

**Figure 1:**
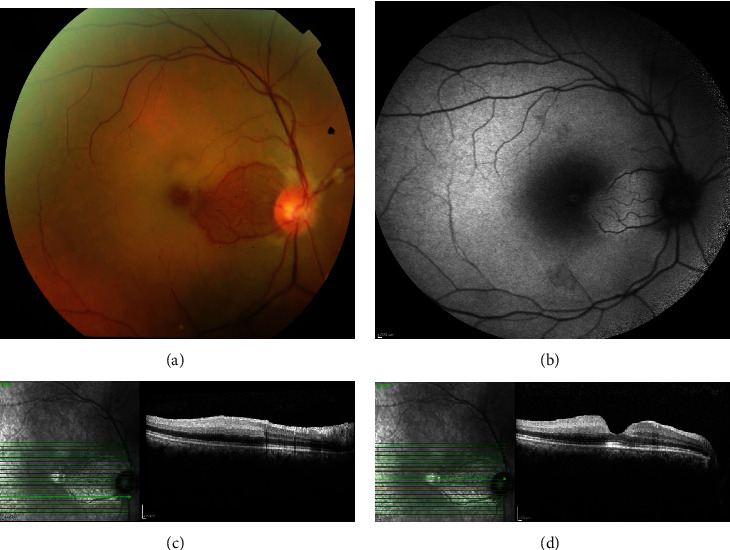
At the first presentation (a). (b) Color fundus and red free photographs of the right eye: diffuse retinal edema except a limited area in papillomacular bundle (c). (d) SD-OCT of the right eye: increased retinal thickness and inner retinal hyperreflectivity in the ischemic areas.

**Figure 2:**
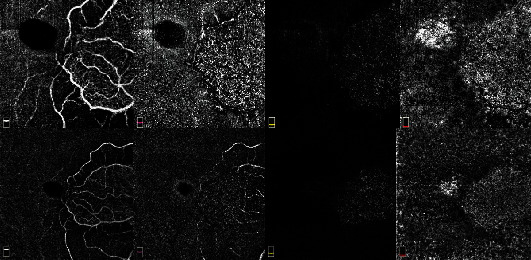
OCT-A of the right eye at first presentation shows no flow in superficial and deep retinal capillary plexuses microvasculature. Only the flow in cilioretinal artery is visible.

A forty-two-year-old man presented with a sudden painless loss of vision in the right eye since three hours ago. Visual acuity was decreased to counting finger in two meters in the right eye with relative afferent pupillary defect. Except, a limited area in papillomacular bundle in the area nourished by patent cilioretinal artery, retinal whiteness, and cherry red spot was prominent (Figures 1(a) and 1(b)). Left eye examination revealed mild retinal veins congestion and optic nerve head hyperemia. In the last night, he consumed a 100 mg film-coated sildenafil tablet (Vizarsin, Krka, d. d., Novo mesto, Slovenia), and twelve hours later, he took another one. The last one was used six hours before visual loss. He was diagnosed with CRAO with cilioretinal artery sparing. After describing the nature of the arterial occlusion for the patient and low chance for vision recovery, although we did not find any emboli in examination, anterior chamber paracentesis was done. Unfortunately, this immediate measures taken to restore the circulation proved futile. In spectral domain optical coherence tomography (SD-OCT), inner retinal hyperreflectivity was obvious (Figures 1(c) and 1(d)) and in optical coherence tomography angiography (OCTA), both superficial and deep capillary retinal plexuses microvasculature were depleted and rendered no flow (Figure 2).

Four weeks later, resolving of retinal edema was obvious in fundus examination of the right eye, and BCVA improved to 20/60 with right head turn. In evaluation for systemic vasculitis and other rheumatologic diseases, cardiologic evaluation for cardiac or carotid vessels pathologies, and neurologic imaging, the patient was found to be healthy. He declared that he had been consuming sildenafil tablets in different doses for the past five years. In the last three years, he consumed Vizarsin 100 mg film-coated tablets, but has never taken two tablets in a short interval.

## 3. Discussion and Conclusion

Here, we report a patient who suffered from sudden and severe vision loss because of CRAO with a recent history of consumption of two 100 mg sildenafil tablets 12 hours apart. He was otherwise healthy and had history of sildenafil consumption for five years, but in this episode, he consumed a high dose of the medication.

Various ocular side effects have been described following the use of sildenafil. These fall into two major categories: (1) those secondary to the weak inhibitory effects of sildenafil on the isoenzyme PDE in the retina such as temporary loss of vision, green/blue tinging of vision, increased sensitivity to light, and blurred vision [5]. These symptoms are mild, dose-dependent, and completely reversible and occur in 3–11% of men taking sildenafil 25–100 mg [6]. (2) Those secondary to vascular events includes anterior ischemic optic neuropathy [7–9], cilioretinal artery occlusion [10], branch retinal artery occlusion [5], central retinal vein occlusion [11], and acute macular neuroretinopathy [12].

Murthy [13] reported a 37-year-old African American woman with a known history of sickle cell anemia and pulmonary arterial hypertension who was receiving treatment with tadalafil, a PDE5 inhibitor, developed bilateral, concurrent CRAO that persisted after exchange transfusion.

Previous studies regarding the sildenafil effect on ocular blood flow have reported inconsistent results. Most of them showed increased choroidal blood flow (CBF) with a lesser effect on the retinal vasculature. This difference may be due to discrepancy in vascular innervation. In this respect, the innervation of the choriocapillaris resembles that of the corpus cavernosum. Theoretically, because sildenafil has a strong systemic vasodilating effect that decreases systemic blood pressure, it can result in decreased CBF. Different balances of these factors among individuals can lead to variable effects on ocular blood flow [14]. In contrast to increased blood flow as a result of vasodilation by the smooth muscle relaxant effects, thrombus formation can have impaired blood flow in the vessel. Vascular insufficiency and stasis are major factors causing the initial formation and progression of thrombosis. High concentrations of cyclic guanosine monophosphate induced by the long-term use of sildenafil are known to mediate the changes in endothelial permeability and finally promote platelet adhesion and enhance thrombus formation [15].

In our case, a patient with a history of sildenafil consumption for years, he had taken two 100 mg sildenafil tablets twelve hours apart, while the standard recommended doses of sildenafil range from 25 to 100 mg three times weekly [14]. The peak plasma level after oral intake occurs at about 60 min with an elimination half-life of 3–5 h [14]. Although the occurrence of CRAO in our patient after sildenafil use may be coincidental, a possible association should not be overlooked. In this case, it seems to be a dose-response and temporal relationship between the sildenafil and the side effect, CRAO, in terms of occurrence within a reasonable amount of time after high dose drug ingestion. Although it cannot be explicitly proven that sildenafil was the cause of CRAO in this patient as other factors could also have been involved in CRAO, the close temporal relationship strongly suggests this.

Our hypotheses for mechanisms that may contribute to the CRAO in this patient include (1) a thrombotic event in artery due to enhanced platelet adhesion as a consequence of long-term sildenafil use or (2) the compressive effect of dilated vein on artery leading to decreased arterial blood flow. At the first presentation, we noted mild retinal veins congestion and optic nerve head hyperemia in the left eye. Such an event may have happened in the right eye and eventually led to CRAO.

CRAO as a sight threatening condition may be a complication associated with the use of sildenafil. Physicians should be aware of this side effect and should seek other alternatives in patients with high risk of developing CRAO. The patients using sildenafil should closely collaborate with the ophthalmologist and appeal to them when any visual symptom is experienced.

## Data Availability

The datasets used and/or analyzed during the current study are available from the corresponding author on reasonable request.

## Abbreviations

PDE: Phosphodiesterase
CRAO: Central retinal artery occlusion
BCVA: Best corrected visual acuity
EDI-OCT: Enhanced depth imaging optical coherent tomography
SD-OCT: Spectral domain optical coherent tomography
CBF: Choroidal blood flow
DCP: Deep capillary plexus
SDP: Superficial capillary plexus.
